# Auditory processing in patients with temporal lobe epilepsy

**DOI:** 10.1016/S1808-8694(15)30995-2

**Published:** 2015-10-19

**Authors:** Juliana Meneguello, Fernando Danelon Leonhardt, Liliane Desgualdo Pereira

**Affiliations:** a MS in Human Communication Disorders, Audiologist; b MS in Otorhinolaryngology/Head and Neck Surgery; c PhD in Human Communications Disorders, Head of the Speech and Hearing Department - UNIFESP. Federal University of São Paulo - Paulista School of Medicine

**Keywords:** temporal lobe, hearing, epilepsy, auditory perception, hearing disorders

## Abstract

Temporal epilepsy, one of the most common presentation of this pathology, causes excessive electrical discharges in the area where we have the final station of the auditory pathway. Both the anatomical and functional integrity of the auditory pathway structures are essential for the correct processing of auditory stimuli.

**Aim:**

to check the Auditory Processing in patients with temporal lobe epilepsy regarding the auditory mechanisms of discrimination from sequential sounds and tone patterns, discrimination of the sound source direction and selective attention to verbal and nonverbal sounds.

**Method:**

eight individuals with temporal lobe epilepsy were assessed, after excluding those with non-confirmed diagnosis or with the focus of discharges not limited to this lobe. The evaluation was carried out through special auditory tests: Sound Localization Test, Duration Pattern Test, Digits Dichotic Test and Non-Verbal Dichotic Test. Their performances were compared to the performances of individuals without neurological diseases (case-control study).

**Results:**

similar performances were observed between patients with temporal lobe epilepsy and the control group regarding the auditory mechanism of sound source direction discrimination. Comparing the other auditory mechanisms assessed, the patients with temporal lobe epilepsy presented worse results.

**Conclusion:**

individuals with temporal lobe epilepsy had more deficits in auditory processing than those without cortical damage.

## INTRODUCTION

Epilepsy is a set of clinical manifestations that may reflect a temporary neural dysfunction - abnormal and excessive electric discharges^1^. Many are the causes for this disorder, such as: pre and post natal infections, traumas, parasitic infestations, intoxication, strokes, genetics, or even unknown causes^2^. According to the WHO (1994), this disease affects from three to five individuals for 1000 of the world population, and in developing countries this figure may reach 15 to 50 for each 1000 inhabitants. Temporal lobe epilepsy is the most common form of the disease, and the one most difficult to control. Data as to its occurrence vary between 50% of epileptic adults and 70 to 80% of teenagers with the disease^1^.

The auditory pathway ends at the temporal lobe (primary and secondary auditory cortex), after going through many central and peripheral structures of the auditory system. Knowing that for a correct analysis and interpretation of the information received through one's hearing (auditory processing) it is necessary to have full anatomical and functional integrity of all these structures and that the electric discharges caused by the crisis may cause neuronal losses in the region where they occur, therefore it has been thought that there may be difficulties in the mental processing of the information received through hearing, and this could impair even further the communication skills of these patients.

The goal of this paper is to check the Auditory Processing of temporal lobe epilepsy patients, as to the mechanisms of: discrimination of sounds in sequence and of tonal patterns, discrimination of the sound source direction, and recognition of verbal and non-verbal sounds in dichotic hearing. Thus, we try to understand the effect of epileptic spells in the auditory processing of individuals with this neuronal disorder, aiming at improving their language rehabilitation process.

## MATERIALS AND METHODS

This study was carried out in the Department of Hearing Disorders of the Federal University of São Paulo - Paulista School of Medicine (UNIFESP-EPM), after being approved by the Research Ethics Committee of the same University (515/00). The assessment started after the individuals read the Information Letter about the study goals and signed the Informed Consent Form to participate in the study. This was designed to be a “case-controlled study”.

35 patients were referred from the Department of Epilepsy of the Neurology Center of the São Paulo University Hospital. Of these, only 13 came to our department for Auditory Processing Assessment. Notwithstanding, five patients were withdrawn from the sample because they had epilepsy spells that were not restricted to the temporal lobe or did not have a confirmed diagnosis of temporal lobe epilepsy. We then assessed eight male and female patients, with ages varying between 22 and 51 years (Group I). All the individuals who took part in the study were diagnosed with Temporal Lobe Epilepsy and had their diagnosis confirmed by the Department of Neurology of the University (Table 1). They all received the proper medication to treat this disease, according to what was necessary in order to reduce the epileptic crisis to the lowest possible frequence and were all right-handed, according to their preferred hand for writing.

**Table 1:** Group I individuals according to gender Males (M) or Females (F), age / in years, location of the lesion, time of spells onset in years, presence of temporal lesion.

Obs.	Gender	Age (in years)	Location of the lesion	Time of onset (in years)	Temporal lesion seen on the CT-Scan
1.	M	22	Temporal Left	7	No
2.	F	51	Temporal Left	37	No
3.	M	32	Temporal Left	14	Yes
4.	F	32	Temporal Left	17	No
5.	M	42	Temporal Right	32	No
6.	M	38	Temporal Left	37	No
7.	F	33	Temporal Left	21	No
8.	F	41	Temporal Right	21	No

The patients underwent a medical interview and later a basic audiologic evaluation, comprising threshold tonal audiometry, speech recognition threshold (SRT), acoustic immitance measures and acoustic reflex thresholds. This assessment was carried out in order to check on the existence of any peripheral alteration that could interfere in the patient's performance in carrying out central behavioral tests. None of the patients showed any alteration that would exclude him/her from the study.

The individuals were then submitted to four behavioral tests in order to assess their auditory processing:

1) Sound Location Test: according to Pereira (1993)^3^, we assessed the identification behavior of each one of the five sound sources in relation to the patient's skull (right side, left side, above, behind, in front of) in a free field. The expected and proper result was the correct identification of at least four directions, and the patients had to state either the right or left side.
2) Recognition Test of the Duration Pattern: according to Musiek^4^, we assessed the identification behavior, by appointing, a sound pattern made up of a series of three short sounds (pure tones in the frequence of 1000Hz) with different inter-stimuli intervals and different durations (short tone, C, of 250ms and, long, L, of 500ms). The stimuli (30 different items for each ear) were presented through the audiometer, in a sound treated booth, at an intensity level of 50dBSL, having the average of hearing thresholds as reference in the frequencies of 500, 1000 and 2000Hz. We considered 83% of correct identification to be normal in the sequences used as stimuli, according to Borges, Corazza, Pereira (1999)^5^.
3) Digits dichotic test - which assesses the identification behavior, through verbal repetition, familiar words presented at a dichotic hearing, in other words, at the same time, a different one for each ear. We used the recorded version according to what Pereira described (1997)^6^, at the Binaural Integration stage. The stimuli (two-syllable digits in the Portuguese Language “quatro”, “cinco”, “sete”, “oito”, and “nove”) were presented through the audiometer, in a sound treated booth, at an intensity level of 50dBSL, having the averages of hearing thresholds as references in the frequencies of 500, 1000 and 2000Hz according to what was proposed by Santos, Pereira, Fukuda (1999)^7^. An error was considered when a word from the item was omitted or incorrectly identified. As normal values, we accepted that each individual would have 95% of correct answers or more in each ear.
4) Non-Verbal Dichotic Test: assesses the identification behavior, by pointing to representative figures, one between two non-verbal sounds presented at the dichotic hearing, in other words, simultaneously, a different one for each ear. We used the recorded version, according to Pereira (1997)^6^. The stimuli were presented through the audiometer, in a sound-treated booth, at an intensity level of 50dBSL, having the average of the hearing thresholds as reference in the frequencies of 500, 1000 and 2000Hz of the better ear. The test was carried out in the three steps, called Free Attention Stage and Guided Hearing Stage to the right and to the left.

At the Free Attention Stage, the patient would freely choose which of the two sounds he/she would identify among the 24 pairs of stimuli presented. At the Right Side Guided Hearing Stage (EDD) the patient should identify only the sound stimuli hear through the right ear and at the Left Side Guided Hearing Stage (EDE) he/she should only identify the sound stimuli heard through the left ear. At the guided hearing stages, 12 pairs of stimuli were presented. As a reference for normality, we accepted the symmetry of responses, according to Ortiz, Pereira, Vilanova (2003)^8^, at the Free Attention Stage and identification ratio above 90% of the stimuli heard at the selected ear in the Guided Hearing Stage.

The procedures used to carry out the tests, as well as the reference values for normality followed those used in the Hearing Processing Outpatient Ward at the Department of Hearing and Speech Sciences of the Federal University of São Paulo.

In order to assess how the mental processing of information received through hearing occurs, we used a set of tests encompassing the different auditory mechanisms described by Guyton & Hall, 1997^9^, which were: identification of tonal patterns, of sound sequencing and sound source direction, and sound inhibition up to 20dB (selective attention). We chose tests that used both verbal and non-verbal stimuli.

The results attained were compared to those from a group of individuals without neurological impairments, called Group II. Thus, four males and six females, with ages matching those individuals from Group I (Table 2), underwent the same evaluation.

**Table 2:** Individuals from Group II, according to gender and age.

Individual	Gender	Age (in years)
1.	Female	16
2.	Male	20
3.	Female	26
4.	Female	25
5.	Male	22
6.	Male	29
7.	Female	41
8.	Male	26
9.	Female	38
10.	Female	47

In order to include Group II individuals, the following criteria were used: no complaints and/or symptoms related to epilepsy or other neurological alteration and presence of responses to threshold tonal audiometry and acoustic immitance within normal limits.

In order to analyze the results, we chose a descriptive statistics approach, calculating the average values of right answers, in percentages, even because the small sample size of individuals with temporal lobe epilepsy made it unfeasible the use of conventional statistical tests.

## RESULTS

Table 3 depicts results for groups I and II, the results attained in the Sound Source Location Test, selected to assess the sound source direction identification mechanism.

**Table 3:** Individuals from Groups I and II, according to the correct answers obtained from the Sound Location Test.

Group	CORRECT 4 correct	ANSWERS 5 correct	Total
Group I	2 (25%)	6 (75%)	8 (100%)
Group II	0 (0%)	10 (100%)	10 (100%)

Tables 4 (Group I) and 5 (Group II) show the data obtained in the Duration Pattern Test, selected to assess the hearing mechanism of sequence sounds discrimination and tonal patterns (temporal ordering).

**Table 4:** Percentage of correct answers by individuals from Group I at the Duration Pattern Test.

Individual	Right Ear	Left Ear
1.	90%	97%
2.	50%	67%
3.	30%	33%
4.	80%	73%
5.	73%	73%
6.	10%	13%
7.	53%	67%
8.	40%	30%

Average of correct answers: 53.3% 56.6%

**Table 5:** Percentage of correct answers per individual from Group II at the Duration Pattern Test.

Individual	Right Ear	Left Ear
1.	97%	90%
2.	87%	93%
3.	87%	77%
4.	100%	100%
5.	97%	100%
6.	100%	100%
7.	67%	80%
8.	100%	97%
9.	57%	60%
10.	60%	60%

Average of correct answers: 85.2% 85.7%

Tables 6 (Group I) and 7 (Group II) depict the data obtained thorough the Digits Dichotic Test, selected to assess the familiar verbal sounds recognition mechanism in dichotic hearing (selective attention for verbal sounds).

**Table 6:** Percentage of correct answers per individual from Group I at the Digits Dichotic Test.

Individual	Right Ear	Left Ear
1.	100%	97,5%
2.	97,5%	97,5%
3.	92,5%	97,5%
4.	92,5%	92,5%
5.	92,5%	72,5%
6.	100%	90%
7.	92,5%	95%
8.	90%	90%

Average of correct answers: 94.6% 91.5%

**Table 7:** Percentage of correct answers per individual from Group II at the Digits Dichotic Test.

Individual	Right Ear	Left Ear
1.	100%	100%
2.	100%	97,5%
3.	100%	100%
4.	100%	100%
5.	100%	100%
6.	100%	100%
7.	95%	100%
8.	100%	100%
9.	100%	95%
10.	97,5%	95%

Average of correct answers: 99.2%98.7%

Tables 8 (Group I) and 9 (Group II) depict the results attained at the Non-Verbal Dichotic Test Free Attention Stage, while Tables 10 (Group I) and 11 (Group II) depict the results obtained by ear requested for the Left and Right Guided Hearing Stages of this same Test, which was used to assess the non-verbal sounds recognition mechanism in dichotic hearing (selective attention for non-verbal sounds).

**Table 8:** Number of correct answers per individual from Group I at the Non-Verbal Dichotic Test - Free Attention Stage.

Individual	Right Ear	Left Ear
1.	09	14
2.	07	17
3.	13	10
4.	11	13
5.	13	09
6.	13	11
7.	09	15
8.	15	09

Average of correct answers: 11.3 12.3

**Table 9:** Number of correct answers per ear, left or right, and per individual from Group II at the Non-Verbal Dichotic Test - Free Attention Stage.

Individual	Right Ear	Left Ear
1.	13	11
2.	09	15
3.	13	11
4.	09	15
5.	15	09
6.	08	16
7.	10	14
8.	11	13
9.	10	13
10.	13	11

Average of correct answers: 11.1 12.8

**Table 10:** Number of correct answers for the requested ear, per individual from Group I at the Non-Verbal Dichotic Test - Right (EDD) and Left (EDE) Sides Guided Hearing Stage.

Individual	EDD Stage Right Ear	EDE Stage Left Ear
1.	12	12
2.	12	12
3.	12	12
4.	08	10
5.	12	12
6.	12	11
7.	12	11
8.	12	12

Average of correct answers: 11.5 11.5

**Table 11:** Number of correct answers for the requested ear, per individual from Group II at the Non-Verbal Dichotic Test - Right (EDD) and Left (EDE) Sides Guided Hearing Stage.

Individual	Right Ear EDD Stage	Left Ear EDE Stage
1.	11	12
2.	12	12
3.	12	12
4.	12	12
5.	12	12
6.	12	12
7.	12	12
8.	12	12
9.	12	12
10.	11	11

Average of correct answers: 11.8 11.9

## DISCUSSION

Individuals with temporal lobe epilepsy whom comprised the Group I had their performances similar to those individuals from normo-hearing comparison group, whom comprised Group II, as to the sound source discrimination mechanism (Table 3).

As we analyzed the results from the Sound Location Test, selected to assess the Sound Source Direction Discrimination mechanism and the sound source location capability, we noticed that (Table 3) 100% of the individuals from Groups I and II had four or more correct answers, in five presentations, in the test application. This same test was able to show how adequate the hearing mechanism of Sound Source Direction Discrimination is and the Binaural Interaction capability of the individuals from both groups studied, and epilepsy did not constitute a factor of differentiation.

In the specialized literature we see that the structures responsible for sound location are the superior olivary complex and the inferior colliculus, located at the Brain Stem^10^, ^11^, ^12^, as well as the auditory cortex^9^. Moreover, there are reports that any loss in the sound source direction discrimination assessed in a free field, suggests problems at the brain stem level. Thus, in patients with temporal lobe epilepsy, it would not be expected to see alterations in the Sound Location Test, shown by disorders in the workings of the Brain Stem.

Bellis (1996) described sound location as a function of binaural interaction, and it reflected the way through which the information coming from each ear interact, in other words, how they are processed together. Alterations in this capability would be justified by an asymmetrical peripheral hearing loss or alterations in the hearing processing that takes place in the Brain Stem^12^. Our research did not lead us to expect alterations in the Tests caused by hearing loss, because all the individuals had tonal thresholds within the normal range.

The literature also has reports of complex sounds being used in sound location tests, such as the one used in the present research study. This type of sound would be more easily located than pure sounds in carrying out this type of test^13^.

The findings of our study are in agreement with those from Shankweiler (1961), who did not see performance differences in those individuals with alterations in the temporal lobe when compared to normal individuals or individuals with alterations in other brain regions^14^; those from Nilsson, Lidén (1976) who saw good performances in those individuals with intracranial lesions in sound location tests^11^; from Abel, Birt, Mclean, (1978), who stated equal results between normal and individuals with temporal lobe lesion in sound locating tests^15^ and those from Pereira (1993) who also did not see worse results in the Sound Location Test in Epileptic children when compared to normal children^3^. Notwithstanding, these findings disagree from the ones attained by other authors who developed a Sound Location Test and observed worse performance from individuals with temporal lobe lesion when attempting to locate sounds contralaterally to the lesion when compared to individuals without brain lesions or those with extra-temporal lesions^13^, ^16^.

Individuals with temporal lobe epilepsy, Group I, presented lower performance than the comparison group of normal-hearing individuals who comprised Group II (Table 4, Table 5, Table 6, Table 7, Table 8, Table 9, Table 10, Table 11) as to the hearing mechanisms of sound discrimination in sequence and tonal patterns (temporal ordering) and verbal and non-verbal sounds in dichotic hearing (selective attention).

In this paper, the performance from Group I and II individuals in the Duration Pattern Test (Tables 4 and 5) was different, and the individuals from Group II (average of about 85% per ear) had average correct answer values higher than the Group I individuals (approximately 55% per ear). Making a comparative analysis of correct answers obtained for the right ear (OD) and the left ear (OE), we saw that the performance of the individuals was similar in Groups I (OD =53.25% and OE= 56.62%) and II (OD = 85.2% and OE=85.7%), and we did not notice response asymmetry between the ears for any of the groups. Group I individuals had higher correct answers percentage variability, while individuals from Group II had lower variability. Temporal ordering difficulty shown by this test may be associated to speech recognition problems.

Thus, we observed that individuals from Group I presented lower performances when compared to those from Group II as discrimination mechanisms for sounds in sequence and tonal patters (temporal ordering) are concerned. Most individuals from Group II had impairments in this mechanism.

We know that through the Duration Pattern Test it is not possible to determine the side of the lesion/dysfunction, since research has shown that results were altered in both ears in individuals with brain lesions, regardless of the side affected^4^, ^5^, ^17^. Musiek (1994) reported that sound temporal processing is necessary for speech decoding and that both brain hemispheres take part in this process, the left side because it is the prevailing side for speech, language and temporal ordering, and the right side for being responsible for the identification of acoustic patterns. Acoustic patterns (frequence, intensity and duration) involve perception and cognitive processes, and the duration process requires greater maturity from the Central Nervous System^4^. Musiek, Baran, Pinheiro (1990) have described it as a higher function, and one more susceptible to hearing cortex pathologies^17^.

In the present study, it also became clear that regardless of the epileptic spell side, the individuals had a deficit in the performance of tasks in this test, in both ears, without differences between them; similar to what has been found in previous studies^4^, ^5^, ^12^, ^17^.

As we analyze the performance of individuals from Groups I and II in assessing the selective attention hearing mechanism for verbal sounds using the Digits Dichotic Test (Tables 6 and 7), we noticed that Group I individuals presented lower rates of correct answers and greater response variability than those from Group II. Six individuals (80%) from Group I presented performances lower than 95% of correct answers in one of the ears, while 100% of the individuals from Group II presented similar values or values above this one (95%) in the test. When we compare correct answers given to the stimuli presented to the right (OD) and left (OE) ears, we noticed that only one individual from Group I (# 5) showed a greater than 10%, difference between the ears, indicating response symmetry in all the other subjects tested. In average values, and based on the responses obtained for each one of the Groups, per ear (Group I: OD = 94.6% / OE = 91.5%; Group II: OD = 99.2% / OE = 98.7%), we can see that an increasingly higher number of individuals from Group I had difficulties in the correct identification of the words heard in the dichotic hearing, specially with the left ear, when compared to the ease with which these words were identified by the individuals in Group II.

The studies found reported a preference of the Left Hemisphere (HE) for the processing of verbal sounds, thus, a certain advantage of the right ear (OD) was observed in this type of task for speech stimuli, using syllables, words, logatomas, phrases or digits^7^, ^10^, ^18^, ^19^, ^20^, ^21^, ^22^, ^23^, ^24^, ^25^, ^26^, ^27^, ^28^, ^29^. Geschiwind, Levitsky (1968) stated that the structural asymmetry of the temporal plane justifies the functional asymmetry observed in this type of task, since they saw that the left temporal bone is bigger than its right side counterpart^30^.

Broadbent (1954), when introducing the study with a dichotic task, also observed that when they reproduced the stimuli presented to both ears, individuals would tend to first answer all the stimulus of one ear and, afterwards, of the other ear^18^. A similar report was made by Satz (1968)^22^. Moreover, there are reports that it is easier for individuals to hear sequential than simultaneous stimuli^18^.

Kimura (1961a, 1961b) stated that the contralateral auditory pathways are more efficient than their ipsilateral counterparts and, therefore, for a dichotic task the stimuli is more easily processed by the contralateral ear to the prevailing brain hemisphere for speech sound processing^10^, ^19^. Other authors describe in their papers that response asymmetry at dichotic tasks is to be expected if the stimuli presented to both ears are simultaneous and of the same duration. If that is not the case, the stimulation works as if it were two monoaural presentations, making the asymmetry disapear^21^, ^31^.

Previous studies have shown that the dichotic stimulation in individuals with epilepsy makes the message processing depend on factors such as the presence or absence of brain lesion and the side in which they have the spell or the lesion, in other words, in the presence of cortical lesion, the preference of information processing would be in the contralateral side to it (effect of the lesion); in its absence, it would occur in the hemisphere ipsilateral to the neuronal discharge (paradoxical effect)^23^, ^25^, ^26^, ^27^. Mazzuchi, Visintini, Magnani, Cattelani, Parma (1985) believed that this processing pattern occurs for competitive information of any nature, that is, auditory or visual, verbal or non-verbal^26^.

The results attained in the present research agree with those from Dibi (1996) and Ortiz, Pereira, Vilanova (2002) when they observed worse results in the group of individuals with epilepsy when compared to normal subjects in the verbal dichotic task test^29^, ^32^. However, these findings disagree from the specialized literature since we did not observe clear right ear advantage in the processing of verbal stimuli - dissyllabic digits - in a dichotic task^7^, ^10^, ^18^, ^19^, ^20^, ^21^, ^22^, ^23^, ^24^, ^25^, ^26^, ^27^, ^28^, ^29^. This may have probably occurred due to a greater ease of the stimuli used in the Portuguese version for this research. Dissyllabic words are more easily processed that monosyllabic, syllables and logatomas, as the ones most frequently used in dichotic hearing in tests carried out in English. Moreover, Muszkat (1989) stated that “consonant-vowel”-type stimuli in dichotic tasks are better to show the hemispheric specialization for language^27^ and Kimura (1961a) reported that only stimuli bearing a certain degree of linguistic difficulty are able to show the response asymmetry at the dichotic stimulation^19^.

Another factor that may have influenced the response of the individuals was the speed at which the stimuli were presented, since the lower the stimuli presentation speed in the dichotic tasks, the less response asymmetry is seen^22^.

We also did not observe the presence of a lesion effect, having seen that the only individual from Group I who presented response asymmetry showed a response pattern very different from what is mentioned in the literature^23^, ^25^, ^26^, ^27^.

The Non-Verbal Dichotic Test allows us to assess the selective attention mechanism in a binaural separation task. In this paper we noticed a difference between groups I and II individuals in the performance of this test in the Free Attention Stage. Although response variability was similar among them, more individuals from Group I presented response variabilities (five individuals - 62.5%) when compared to Group II, in which only 40% of the individuals presented such result (Tables 8 and 9). Notwithstanding, we did not observe any advantage of one ear to the other in none of the Groups as far as the average value of correct answers for both right and left ear were concerned (Group I = OD 11.3 /OE 12.3; Group II = OD 11.1 /OE 12.8). Thus, we observed that more individuals from Group I presented asymmetrical responses, showing a worse performance when compared to those from Group II as to the selective attention mechanism for non-verbal sounds at the free attention stage.

In the Left and Right Guided Hearing Stages, there was no difference in the performance of the groups, in general. In none of the groups we observed a predominance of stimuli recognition presented to one of the ears. However, one individual from Group I (number 4), presented discrepant results in these stages of the Test, moving away from the responses of the remaining subjects (Tables 10 and 11). In analyzing test responses of guided hearing of non-verbal sounds, at least seven of the eight individuals from Group I with temporal lobe epilepsy had some difficulty either at the free attention stage or in the guided hearing stage. In the GII, the comparison group without evidences of neurological damage, we noticed improper responses from four of the then people assessed. We may state that there were more improper responses in Group I than in Group II.

Data from the literature show that a number of studies carried out with dichotic hearing for non-verbal sounds used tonal, musical and/or melodic stimuli^26^, ^33^, ^35^, ^36^. Herrero, Hillix (1990) used phrases as stimuli in their study, however considered their prosodic content variable^31^. Studies using similar stimuli to the ones used here assessed normal and epileptic individuals with the non-verbal test^8^, ^29^.

Studies carried out with normal individuals show a clear preference for the Left Ear for the processing of non-linguistic sounds^26^, ^31^, ^32^, ^33^, ^35^, ^36^, ^37^, except if the tests are applied to musicians^32^, ^35^. However, in Brazil, Ortiz, Pereira, Vilanova (2003) did not state this type of occurrence, they did observe a response symmetry at the Test stage which freed the individuals to respond the stimuli presented to any of two ears and when compared to the stages that forced the individuals response to the right ear (OD) or to the left ear (OE)^8^. Also Spellacy (1969) apud Spellacy, Blumstein (1970) did not see any advantage of one ear over the other when carrying out environmental stimuli^38^.

The OE advantage for non-linguistic sound processing indicates a preference for the right hemisphere (HD)^25^ for such dichotic test. Kimura (1963) had already reported a preference for the Left Hemisphere (HE) in the processing of information for verbal sounds^20^ in dichotic hearing. Thus, the processing of non-verbal sounds would be generally carried out by the HD in those individuals without musical experience, differently from the analytical process that the HE carries out for verbal stimuli^32^. Now, in musicians, the musical stimuli processing happens as it does for linguistic stimuli^32^, ^35^. However, Mazzuchi, Parma, Cattelani (1981) admitted that there is no consensus about the processing of this type of auditory information^37^.

Kimura (1961a; 1961b) reported that the contralateral pathways are more efficient than their ipsilateral counterparts and, therefore, the dichotic stimulation responses are asymmetrical, with advantages for the ear contralateral to the predominant hemisphere in auditory stimulus processing. Moreover, he also described that the very easy stimuli would not cause this asymmetry in their processing^10^, ^19^.

Authors who studied patients with epilepsy saw that patients with proven cerebral lesion presented the “lesion effect”, in other words, a predominance of the contralateral hemisphere to the lesion in sound processing, regardless of it being verbal or not. In the patients that did not show any lesions in their CT-Scan, the specialized literature reported 26 a predominance of stimuli processing by the hemisphere ipsilateral to the spells side. Other studies^23^, ^25^, ^27^ had already shown these effects in the processing of verbal stimuli.

The studied carried out by Ortiz, Pereira, Vilanova (2002) compared the performance of individuals with partial epilepsy spells to those with generalized episodes in regards of the dichotic task with non-verbal sounds. They saw that both Groups presented similar performances, which were worse than that of normal individuals. This study also reported the lack of ear advantage in the nonverbal stimuli^29^.

The results attained in this paper corroborate those from Ortiz, Pereira, Vilanova (2003) when they analyzed the Free Attention Stage of Group II individuals, since the author also observed response symmetry in normal individuals in this Test stage^8^, and those from Ortiz, Pereira, Vilanova (2002) who noticed a worse performance in epileptic individuals in the Non-Verbal Dichotic Test when compared to normal individuals and lack of ear advantage in the processing of non-verbal processing^29^. However, they disagree when the author mentioned a difficulty the epileptic individuals have in lateralizing their attention to one of the ears, since of those group I individuals, only one of them presented a high number of errors in the Guided Hearing Test. This may be due to the age difference among the individuals of both studies, having seen that the other was carried out with epileptic children.

The findings of this paper are also in agreement with those from Spellacy (1969) apud Spellacy, Blumstein (1970) which did not see differences between the ears when they applied a test using environmental stimuli^38^.

Notwithstanding, these findings are in disagreement from others that did report left ear advantages towards the processing of non-verbal stimuli, such as tonal sequences, melodies, prosodic characteristics and sound effects^26^, ^31^, ^32^, ^34^, ^35^, ^36^, ^37^. The difference observed between the results of this paper and those from the surveyed literature may be due to factors such as different testing conditions, stimulus type and presentation mode^37^, ^39^.

As we analyze the presence of a lesion effect or the paradoxical effect, we noticed that despite the average symmetry of responses between the ears in the Free Attention Stage in both groups, in Group I individuals who presented response asymmetry there was an agreement with the findings reported in the literature, that is, the individual who presented a lesion in the cortical hemisphere presented the “lesion effect”, while the other individuals with response asymmetry presented the “paradoxical effect”^23^, ^25^, ^26^, ^27^.

In the present study we have noticed that the temporal lobe was a factor that differentiated sound processing and the individuals with this disorder presented a loss in the analysis of their verbal and non-verbal sounds received through hearing, when compared to subjects without cortical alterations. The presence of alterations, either structural or functional in the temporal region caused impairment for these individuals, since this cortical region is responsible for the processing of acoustic information. However, the reduced sample of this study made impossible some analyzes types, such as the use of medication (mono or polytherapy), frequence of spells, age of onset and type of crisis, besides the use of statistical tests to compare the responses when of the assessment o auditory processing between the Groups. Later studies with a larger number of patients that would allow us to control these variables would broaden the understanding of how the mental processing of auditory information happens in individuals with temporal lobe cortical alterations.

## CONCLUSION

The results of this research, which assessed the Hearing Processing in individuals with temporal lobe epilepsy, show that the affected individuals had a similar performance to those age matching controls without cortical damage, as to their sound source direction discrimination hearing mechanism (sound source location), and greater loss in the processing of hearing received information for sounds in sequence and tonal patterns (temporal ordering) discrimination mechanism and the recognition of familiar verbal sounds and non-verbal sounds in dichotic hearing (selective attention).
